# Discovery and characterization of an F_420_-dependent glucose-6-phosphate dehydrogenase (Rh-FGD1) from *Rhodococcus jostii* RHA1

**DOI:** 10.1007/s00253-016-8038-y

**Published:** 2016-12-13

**Authors:** Quoc-Thai Nguyen, Gianluca Trinco, Claudia Binda, Andrea Mattevi, Marco W. Fraaije

**Affiliations:** 1 Molecular Enzymology Group, Groningen Biomolecular Sciences and Biotechnology Institute, University of Groningen, Nijenborgh 4, 9747 AG Groningen, The Netherlands; 2 Scuola Universitaria Superiore IUSS Pavia, Piazza della Vittoria 15, 27100 Pavia, Italy; 3 Faculty of Pharmacy, University of Medicine and Pharmacy, Ho Chi Minh City, 41 Dinh Tien Hoang Street, Ben Nghe Ward, District 1, Ho Chi Minh City, Vietnam; 4 Membrane Enzymology Group, Groningen Biomolecular Sciences and Biotechnology Institute, University of Groningen, Nijenborgh 4, 9747 AG Groningen, The Netherlands; 5 Department of Biology and Biotechnology, University of Pavia, Via Ferrata 1, 27100 Pavia, Italy

**Keywords:** *Rhodococcus*, F_420_, Deazaflavoenzymes, Glucose-6-phosphate dehydrogenase

## Abstract

Cofactor F_420_, a 5-deazaflavin involved in obligatory hydride transfer, is widely distributed among archaeal methanogens and actinomycetes. Owing to the low redox potential of the cofactor, F_420_-dependent enzymes play a pivotal role in central catabolic pathways and xenobiotic degradation processes in these organisms. A physiologically essential deazaflavoenzyme is the F_420_-dependent glucose-6-phosphate dehydrogenase (FGD), which catalyzes the reaction F_420_ + glucose-6-phosphate → F_420_H_2_ + 6-phospho-gluconolactone. Thereby, FGDs generate the reduced F_420_ cofactor required for numerous F_420_H_2_-dependent reductases, involved e.g., in the bioreductive activation of the antitubercular prodrugs pretomanid and delamanid. We report here the identification, production, and characterization of three FGDs from *Rhodococcus jostii* RHA1 (Rh-FGDs), being the first experimental evidence of F_420_-dependent enzymes in this bacterium. The crystal structure of Rh-FGD1 has also been determined at 1.5 Å resolution, showing a high similarity with FGD from *Mycobacterium tuberculosis* (Mtb) (Mtb-FGD1). The cofactor-binding pocket and active-site catalytic residues are largely conserved in Rh-FGD1 compared with Mtb-FGD1, except for an extremely flexible insertion region capping the active site at the C-terminal end of the TIM-barrel, which also markedly differs from other structurally related proteins. The role of the three positively charged residues (Lys197, Lys258, and Arg282) constituting the binding site of the substrate phosphate moiety was experimentally corroborated by means of mutagenesis study. The biochemical and structural data presented here provide the first step towards tailoring Rh-FGD1 into a more economical biocatalyst, e.g., an F_420_-dependent glucose dehydrogenase that requires a cheaper cosubstrate and can better match the demands for the growing applications of F_420_H_2_-dependent reductases in industry and bioremediation.

## Introduction

**Fig. 1:**
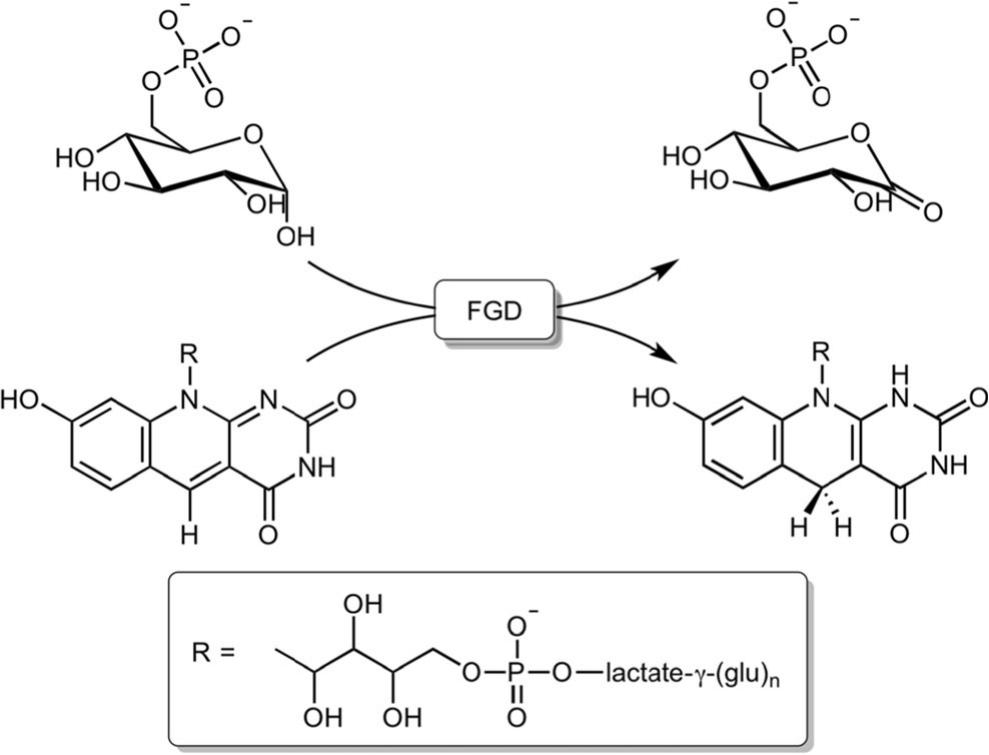
Reaction catalyzed by F_420_-dependent dehydrogenase (*FGD*). Glucose-6-phosphate is oxidized into 6-phosphogluconolactone by FGD concomitantly with the formation of the reduced F_420_ coenzyme, which is subsequently employed by various F_420_H_2_-dependent reductases

The unusual cofactor F_420_, a 7,8-didemethyl-8-hydroxy-5-deazariboflavin, was originally discovered in various archaea (Cheeseman et al. 1972) (Fig. 1). It was demonstrated that in both methanogenic and nonmethanogenic archaea, F_420_ represents a central catabolic redox cofactor involved in the oxidation of energy sources (e.g., H_2_ and formate) (Jacobson et al. 1982; Vitt et al. 2014; Tzeng et al. 1975a; Wood et al. 2003) and the reduction of cofactors such as NADP^+^ and tetrahydromethanopterin (Tzeng et al. 1975b; Warkentin et al. 2001; Hartzell et al. 1985; Aufhammer et al. 2005). In recent years, it has become clear by genome sequence analyses and biochemical studies that the deazaflavin cofactor is also utilized by numerous enzymes in actinobacteria, including *Mycobacterium tuberculosis* (Mtb)—the notorious causative agent of tuberculosis (Daniels et al. 1985). In actinomycetes, F_420_ was found to be involved in several important processes such as biosynthesis of antibiotics in *Streptomyces* spp. (e.g., tetracycline, lincosamide, and aminoglycoside) (Wang et al. 2013; Coats et al. 1989; Li et al. 2009a), degradation of coumarin derivatives (e.g., carcinogenic aflatoxins) (Taylor et al. 2010; Lapalikar et al. 2012b; Ahmed et al. 2015), and other aromatic compounds (e.g., picrate and related compounds) (Ebert et al. 1999; Heiss et al. 2002; Jirapanjawat et al. 2016). For mycobacteria, there is a compelling evidence that F_420_ is essential to render the bacilli persistent in hostile and challenging environments, such as anaerobic conditions, and oxidative and nitrosative stress (Hasan et al. 2010; Gurumurthy et al. 2013; Purwantini and Mukhopadhyay 2009). Interestingly, in vivo activation of the novel antitubercular nitroimidazole prodrugs—such as pretomanid (PA-824), delamanid (OPC-67683), and TBA-354—strictly requires a selective reduction of these prodrugs facilitated by an F_420_H_2_-dependent reductase (Stover et al. 2000; Matsumoto et al. 2006; Denny 2015). Owing to the newly discovered mode of action, these nitroimidazole compounds are highly promising as they exhibit no cross-resistance with the current front-line antitubercular drugs in vitro and even exert activity on non-replicating tubercle bacilli (Stover et al. 2000; Matsumoto et al. 2006; Singh et al. 2008). Delamanid (OPC-67683) was recently clinically approved for multidrug-resistant tuberculosis whereas pretomanid (PA-824) and TBA-354 are currently in phase III and phase I clinical trials, respectively (Tasneen et al. 2015).

The discovery of this novel antimycobacterial class of drugs is attracting an increasing interest in F_420_-dependent enzyme research (Taylor et al. 2013). Due to the unique redox potential (−340 mV) of F_420_, which is lower than that of FAD (−220 mV) and even of the classical hydrogen carrier NAD(P)^+^ (Jacobson and Walsh 1984; de Poorter et al. 2005), F_420_H_2_-dependent enzymes are capable of catalyzing hydrogenation of a wide range of organic compounds which are otherwise recalcitrant to reductive activation such as enones (Taylor et al. 2010; Lapalikar et al. 2012b; Lapalikar et al. 2012a) and imines (Coats et al. 1989; Li et al. 2009a; Li et al. 2009b) in various heterocycles (Schrittwieser et al. 2015). These enzymes thus hold the promise of being highly valuable in industrial biotechnology and bioremediation, and can be exploited as a complement to the available toolboxes for asymmetric chemical synthesis (Taylor et al. 2013; Greening et al. 2016; Ney et al. 2016).

As most bacterial F_420_-dependent enzymes are involved in catalyzing reductions, several F_420_-dependent dehydrogenases have evolved with the purpose to maintain a cytosolic reservoir of reduced F_420_ (F_420_H_2_). In mycobacteria and other actinomycetes, glucose-6-phosphate dehydrogenases (FGDs) seem to be the main producer of F_420_H_2_, by catalyzing the reaction F_420_ + glucose-6-phosphate → F_420_H_2_ + 6-phosphogluconolactone (Fig. 1). FGD was first identified in *Mycobacterium smegmatis* and subsequently in other actinomycetes, including Mtb (Purwantini and Daniels 1996; Purwantini et al. 1997; Purwantini and Daniels 1998). Since the identification of the first FGD two decades ago in Daniels’ lab (Purwantini and Daniels 1996), only two FGDs from actinomycetes, namely *M. smegmatis* and Mtb, have been characterized in detail (Bashiri et al. 2007; Bashiri et al. 2010). These two FGDs share 37% sequence similarity and belong to an F_420_-dependent enzyme subgroup within the luciferase-like hydride transferase family. The affinity of both enzymes for F_420_ and glucose-6-phosphate (G6P) falls in a comparable range, facilitating the release of the resulting reduced cofactor to be consequently exploited by downstream F_420_H_2_-dependent enzymes. Heterologous expression in *Escherichia coli* of both FGDs was found to be troublesome, often resulting in formation of inclusion bodies. Structural characterization of an FGD from Mtb has been recently described (Bashiri et al. 2008).

Rhodococci are high G+C content, Gram-positive aerobic, non-sporulating actinomycetes of high biotechnological and environmental importance due to their ability to catalyze an array of unique enzymatic reactions (van der Geize and Dijkhuizen 2004). A recent bioinformatic study suggested that *Rhodococcus jostii* RHA1 is among the actinomycetes that carry the largest number of F_420_-dependent enzymes. It was predicted to possess at least 104 deazaflavoenzymes (Selengut and Haft 2010). Nevertheless, up to date, there is no experimental evidence for the presence of deazaflavoenzymes in *R. jostii* RHA1. Therefore, in this work, we aimed to (1) verify the existence of FGDs in *R. jostii* RHA1 (Rh-FGD) by heterologous expression of putative FGD-encoding genes in *E. coli*, (2) characterize the catalytic properties of the identified enzyme(s), and (3) obtain and analyze the crystal structure of a Rh-FGD.

## Materials and methods

### Expression and purification of Rh-FGD1 in *E. coli*

*R. jostii* RHA1 was grown in lysogeny broth (LB) at 30 °C; after which, genomic DNA was extracted using the GenElute Bacterial Genomic DNA kit from Sigma. Three putative *fgd* genes, RHA1_RS43115, RHA1_RS10755, and RHA1_RS43570, were amplified from *R. jostii* RHA1 genomic DNA using Phusion High-Fidelity DNA polymerase (Thermo Scientific) and the corresponding pairs of primers as listed in Table 1. The purified PCR products (100–200 ng) were treated with 0.5 U *Taq* polymerase (Roche) and 0.75 mm dATP by incubation at 72 °C for 15 min to introduce the 3′-A overhangs. The resulting insert DNA fragments were ligated into the pET-SUMO vector according to the instruction manual of the Champion pET SUMO expression system (Invitrogen). The construction of the Rh-FGD1 mutants K197N, K258N, and R282Q was done by using the QuikChange® mutagenesis method with primers (Table 1) designed by the web-based QuikChange® Primer Design Tool (Agilent Technologies) and the pET-SUMO-RHA1_RS43115 plasmid as template. All constructs were confirmed by sequencing.

**Table 1:** List of primers used in this study

*fgd* genes	Forward primers (5′–3′)	Reverse primer (5′–3′)
RHA1_RS43115 (Rh-FGD1)	ATGGTGATCAAGTTCGGGTAC	TCATGCGAGCCCTCGCAG
RHA1_RS10755 (Rh-FGD2)	CTACCCCCGCAGCCG	ATGGCCCACGAACTCAAGC
RHA1_RS43570 (Rh-FGD3)	ATGACACAGCAGTTAAAGCTC	TCAGCCCAGGGCACG
RHA1_RS43115-K197N	TACAACTCCATGCC ATT ACCGGACGTGCAGATG	CATCTGCACGTCCGGT AAT GGCATGGAGTTGTA
RHA1_RS43115-K258N	TGACACCGGAGCAG AAT CATTCGATCGACGATC	GATCGTCGATCGAATG ATT CTGCTCCGGTGTCA
RHA1_RS43115-R282Q	CAGGTGGCGAAG CAG TGGATCGTGGCG	CGCCACGATCCA CTG CTTCGCCACCTG

The mutation sites were indicated as underlined oligonucleotides

Proteins were initially expressed in *E. coli* BL21(DE3), grown in Terrific broth containing 50 μg/mL kanamycin, 1% (*w*/*v*) glucose, and induced with 1 mm isopropyl β-D-1-thiogalactopyranoside (IPTG) at 24 °C when the cells reached OD_600_ ~0.7–0.8. To overcome the insolubility of the overexpressed proteins in *E. coli* BL21(DE3), the expression hosts were changed to *E. coli* C41(DE3) (Lucigen) for both the wild-type and mutant Rh-FGDs. The culture conditions were kept the same as for *E. coli* BL21(DE3), except for the addition of 0.2% (*w*/*v*) glucose. The cells were grown until late stationary phase and harvested by centrifugation at 4600×*g* for 10 min (Beckman–Coulter JA-10 rotor, 4 °C). Cells were resuspended in lysis buffer (50 mm KPi pH 7.8, 400 mm NaCl, 100 mm KCl, 10% (*v*/*v*) glycerol, 1 mm β-mercaptoethanol, 20 mm imidazole) and disrupted by sonication using a VCX130 Vibra-Cell (Sonics & Materials, Inc., Newtown, USA) at 4 °C (5 s on, 10 s off, 70% amplitude, total of 5 min). Following centrifugation at 20000×*g* for 45 min (Beckman–Coulter JA-25.5 rotor, 4 °C) to remove unbroken bacteria and cellular debris, the supernatant was applied onto a 5-mL HisTrap HP column (GE Healthcare) pre-equilibrated in the same buffer. The recombinant enzyme with the His-SUMO tag was eluted with a gradient from 20 to 500 mm imidazole in the same buffer. Fractions containing the pure enzyme as indicated by SDS-PAGE and FGD activity assay were pooled, desalted to remove imidazole, and concentrated in a 30-kDa MWCO Amicon (Milipore) centrifugal filter unit. Protein concentration was estimated using the Waddell’s method (Waddell 1956).

To obtain the native enzyme, the His-SUMO tag was cleaved by incubating with 1% (mol/mol) SUMO protease (Invitrogen) for 2 h at 4 °C. The His-SUMO tag, uncleaved protein, and SUMO protease were removed by applying the cleavage mixture onto a second HisTrap column. The native enzyme was concentrated and finally purified through a Superdex 200 10/300 GL (GE Healthcare) in 10 mm HEPES pH 7.5, 100 mm NaCl, 10% (*v*/*v*) glycerol, 1 mm β-mercaptoethanol prior to crystallization experiments.

### Thermostability

Analysis of Rh-FGD1 thermostability was based on the unfolding temperature, *T*_m_, determined by the use of the *Thermofluor*® technique (Pantoliano et al. 2001) with a Bio-Rad C1000 Touch Thermal Cycler (Bio-rad Laboratories, Inc.) in 96-well plates. Each well had a final volume of 25 μL containing 1.6 μm Rh-FGD, 5 × SYPRO Orange (Invitrogen), buffers, and/or additives. The protein start buffer was exchanged to 50 mm KPi pH 7.8 and 150 mm NaCl for the buffer screen and to 50 mm KPi pH 7.8, 500 mm NaCl, and 100 mm KCl for the additive screen. The compositions of the buffers and additives are described in Boivin et al. (Boivin et al. 2013).

### Spectrophotometric assay for FGD activity

FGD activity was routinely monitored by following the reduction of F_420_ at 420 nm, 25 °C, and pH 7.5 using an absorption coefficient ε_420 nm_ of 41.4 mm ^−1^ cm^−1^ (Eirich et al. 1978; Purwantini et al. 1992) in a V-650 spectrophotometer from Jasco (IJsselstein, The Netherlands). F_420_ was isolated from *M. smegmatis* as previously described (Bashiri et al. 2010; Isabelle et al. 2002) (*M. smegmatis* mc^2^ 4517 and the plasmid pYUBDuet-FbiABC were kind gifts from Dr. G. Bashiri, the University of Auckland, New Zealand). The assay mixture typically contained 50 mm Tris/HCl pH 7.5, 300 mm NaCl, 1 mm β-mercaptoethanol, 1 mm EDTA, 100 nm enzyme, 20 μm F_420_, and 1 mm glucose-6-phosphate (G6P) in a final volume of 500 μL. For steady-state kinetics, 10 nm enzyme was used in the same buffer except for the experiments with glucose that were performed with 500 nm enzyme. Kinetic data were analyzed using nonlinear regression to the Michealis–Menten equation using GraphPad Prism v. 6.0 (GraphPad Software Inc., La Jolla, CA, USA). For the pH optima determination, the reactions contained 40 mm Britton-Robinson buffer (Britton and Robinson 1931), 100 nm enzyme, and 20 μm F_420_ and were initiated by adding 1 mm G6P. In the experiments, enzyme activity was monitored at 401 nm (an isosbestic point of F_420_; ε_401 nm_ = 25 mm ^−1^ cm^−1^) (Jacobson et al. 1982; DiMarco et al. 1990) for 5 min.

### Substrate profiling

Alternative phosphate-sugar substrates for FGD were screened in a SynergyMX microplate spectrophotometer (BioTek) using 96-well plates with clear bottom. The reaction mix (200 μL) contained 100 nm enzyme, 10 mm substrate, and 29.6 μm F_420_ in the same buffer as described in the general spectrophotometric assay. The tested compounds for substrate profiling were d-glucose, d-mannose-6-phosphate, d-fructose-6-phosphate, α-d-glucose-1-phosphate, α-d-galactose-1-phosphate, and d-glucosamine-6-phosphate. The absorbance of F_420_ at 420 nm was monitored in intervals of 45 s for 1 h.

### Crystallization, X-ray data collection, and structural determination of Rh-FGD1

Native Rh-FGD1 crystals were obtained using the sitting-drop vapor diffusion technique at 20 °C by mixing equal volumes of 9.0 mg/mL protein in 10 mm HEPES pH 7.5, 10% (*v*/*v*) glycerol, 100 mm NaCl, 1 mm β-mercaptoethanol and of the mother liquor containing 0.16 m ammonium sulfate, 0.08 m sodium acetate pH 4.6, 20% (*w*/*v*) PEG 4000, and 20% (*v*/*v*) glycerol. X-ray diffraction data were collected at the PXI and PXIII beamlines of the Swiss Light Synchrotron in Villigen, Switzerland (SLS), and at the ID23-1 beamline of the European Synchrotron Radiation Facility in Grenoble, France (ESRF). Image integration and data scaling were processed with MOSFLM (Battye et al. 2011) and programs of the CCP4 suite (Winn et al. 2011). Detailed data processing statistics are shown in Table 2. The Rh-FGD1 structure was initially solved by molecular replacement using MOLREP (Vagin and Teplyakov 2010) using the coordinates of FGD1 from *M. tuberculosis* (PDB ID code 3B4Y) (Bashiri et al. 2008) as the search model devoid of all ligands and water molecules. Model building and structure analysis was carried out with COOT (Emsley and Cowtan 2004) whereas alternating cycles of refinement was performed with REFMAC5 (Murshudov et al. 1997). Figures were created by CCP4mg (McNicholas et al. 2011); atomic coordinates and structure factors were deposited in the Protein Data Bank under the PDB ID code 5LXE.

**Table 2:** Data collection and refinement statistics

PDB ID Code	5LXE
Space group	*P*2_1_2_1_2_1_
Resolution (Å)	1.47
*a, b, c* (Å)	81.4, 88.1, 88.8
*R*_sym_^a,b^ (%)	5.0 (55.0)
Completeness^b^ (%)	98.7 (96.9)
Unique reflections	106,774
Redundancy^b^	3.9 (3.1)
I/σ^b^	10.9 (1.7)
Number of atoms	
Protein	4933
Sulfate/glycerol/water	2 × 5/2 × 6/571
Average B value for all atoms (Å^2^)	25.0
*R*_cryst_^b,c^ (%)	16.2 (27.1)
*R*_free_^b, c^ (%)	18.5 (25.1)
Rms bond length (Å)	0.021
Rms bond angles (°)	2.02
Ramachandran outliers	0

^a^ *R*_sym_ = ∑|I_*i*_ − <I > |/∑I_*i*_, where I_*i*_ is the intensity of *i* ^th^ observation and I is the mean intensity of the reflection

^b^Values in parentheses are for reflections in the highest resolution shell

^c^ *R*_cryst_ = ∑|*F*_obs_ − *F*_calc_|/∑|*F*_obs_| where *F*_obs_ and *F*_calc_ are the observed and calculated structure factor amplitudes, respectively. *R*_cryst_ and *R*_free_ were calculated using the working and test sets, respectively

## Results

### Expression and purification of Rh-FGDs in *E. coli*

Three genes encoding putative homologs of Mtb-FGD (accession number KBJ40183) (Bashiri et al. 2007; Bashiri et al. 2008) were identified by BLAST: RHA1_RS43115 (WP_011600337.1), RHA1_RS10755 (WP_011595003.1), and RHA1_RS43570 (WP_011600440.1) (with 84, 84, and 83% sequence identity to Mtb-FGD1, respectively). These genes were amplified from *R. jostii* RHA1 genomic DNA, cloned into the pET-SUMO vector, and expressed in *E. coli* C41(DE3) as N-terminal SUMO-hexahistidine-fused proteins using IPTG as an inducer. The cultivation conditions were optimized for the production of the soluble and active proteins, resulting in a 48-h growth at 24 °C with 1 mm IPTG in Terrific broth as the most effective condition. By testing the cell extracts containing all three different proteins (RHA1_RS43115, RHA1_RS10755, and RHA1_RS43570 referred to as Rh-FGD1, Rh-FGD2, and Rh-FGD3, respectively), it was found that they all exhibit FGD activity. Rh-FGD1 and Rh-FGD2 exhibited comparable specific activity whereas Rh-FGD3 was >20-fold less active. We focused our exploration on the best expressed FGD, namely Rh-FGD1. Typically, approximately 80 mg of pure Rh-FGD1 was obtained from 1 L of TB culture. Furthermore, it is worth noting that Rh-FGD1 is flanked by genes putatively encoding a 6-phosphogluconate dehydrogenase and a G6P isomerase. This strongly suggests that Rh-FGD1 is indeed a glucose-6-phosphate dehydrogenase.

### Characterization of Rh-FGD1

#### pH optimum

**Fig. 2:**
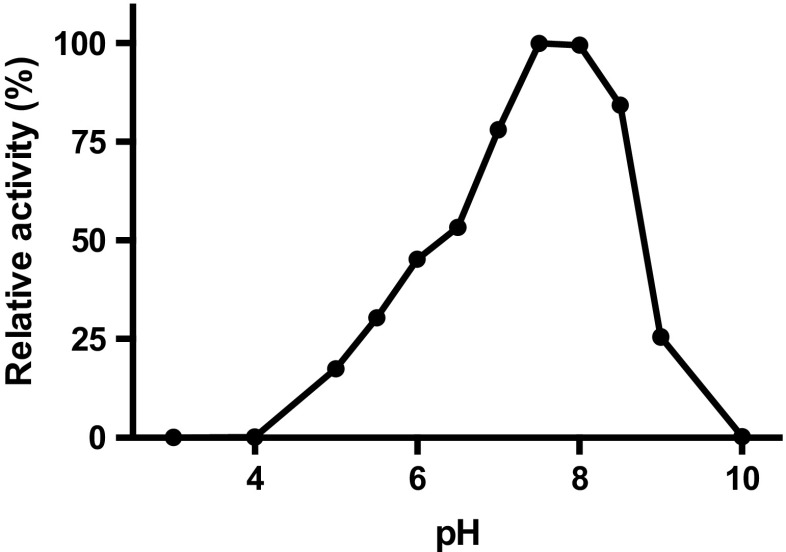
Effect of pH on Rh-FGD1 activity. The reaction contains 40 mm Britton–Robinson buffer, 100 nm Rh-FGD1, 20 μM F_420_, and 1.0 mm G6P, and activity was monitored by following the absorbance at 401 nm (isosbestic point of F_420_) for 300 s at 25 °C

Rh-FGD1 displayed an optimum for activity on glucose-6-phosphate at pH 7.5–8.0 (Fig. 2); this is somewhat similar to the FGDs from Mtb [6.5–7.0 (Bashiri et al. 2008)] and from *M. smegmatis* [two separate pH optima: 6.0 and 8.0 (Purwantini and Daniels 1996)]. For further studies on Rh-FGD1, pH 7.5 was chosen to monitor FGD activity.

#### Substrate profiling

Rh-FGD1 is strictly dependent on F_420_ as coenzyme. The enzyme did not show any significant activity when NAD^+^, NADP^+^, FAD, or FMN was used as alternative electron acceptors. Rh-FGD1 was also found to be highly specific for G6P as electron donor. All tested alternative phosphate-sugars displayed significantly lower activity when compared to G6P. 10 mm d-mannose-6-phosphate, d-fructose-6-phosphate, and d-glucosamine-6-phosphate reached only 1.1, 4.8, and 2.8% of the rate obtained with 1 mm G6P, respectively. The free anomeric carbon C1 of the sugar is crucial for the dehydrogenation as no detectable FGD activity was observed with α-d-glucose-1-phosphate and α-d-galactose-1-phosphate. Rh-FGD1 accepted d-glucose as substrate, although with very low catalytic activity.

#### Thermostability

**Fig. 3:**
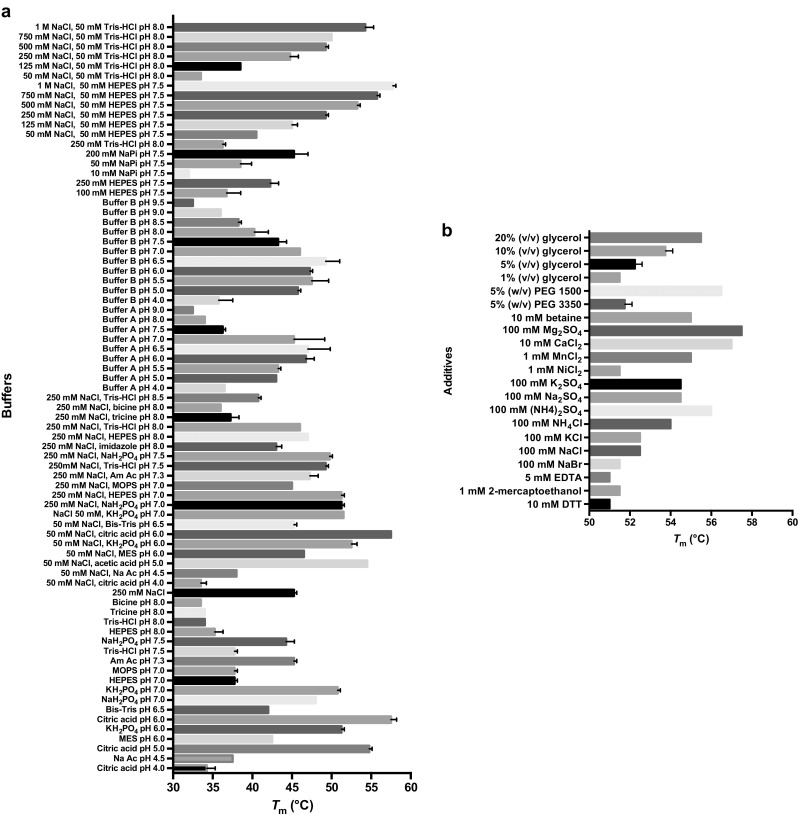
Melting temperatures of Rh-FGD1 in different buffer (**a**) and additive (**b**) conditions measured by the *Thermofluor®* technique. Buffers were used at a concentration of 100 mm unless otherwise indicated. The *error bars* represent SD from the three replicates. *Buffer A* succinic acid/ NaH_2_PO_4_/ glycine = (2:7:7). *Buffer B* citric acid/ CHES/ HEPES = (2:4:3). *Ac* acetate, *Am* ammonium, *DTT* dithiothreitol

The thermostability of Rh-FGD1 was evaluated by determining apparent melting temperatures (*T*_m_) using the *Thermofluor®* technique (Pantoliano et al. 2001). This revealed that Rh-FGD1 represents a stable enzyme, exhibiting *T*_m_ values above 35 °C in most common buffer systems (Fig. 3). The best stabilizing buffers were HEPES, citrate, and phosphate. Several additives were found to have significant effects on the thermostability of Rh-FGD1. NaCl, glycerol, and divalent cations (e.g., Mg^2+^, Ca^2+^, and Mn^2+^) exerted marked effects, resulting in *T*_m_ values of above 55 °C. The stabilizing effect of NaCl depends greatly on its concentration: an increase in NaCl concentration from 50 mm to 1 m (in either HEPES or Tris/HCl) drastically elevates the *T*_m_ by around 20 °C. Based on these findings, we typically stored Rh-FGD1 in a phosphate-based buffer with both NaCl and glycerol as additives. Remarkably, the enzyme can retain >90% of its activity after 1 year when being stored at −80 °C.

#### Steady-state kinetics

For determining the steady-state kinetic parameters with F_420_ and glucose-6-phopshate as substrate, Rh-FGD1 activity was monitored following the decrease in absorbance at 420 nm associated with the reduction of F_420_. The kinetic data did fit well when using the Michaelis–Menten kinetic model. The kinetic parameters for the natural substrates G6P and F_420_ were determined (Table 3) by keeping one of the substrates constant (F_420_ at 20 μm or G6P at 2.0 mm, respectively), while varying the other substrate concentration. The apparent *K*_m_ values for G6P and F_420_ are 0.31 mm and 3.8 μm, respectively. The *K*_m_ value for F_420_ is very similar to that observed with FGDs from Mtb (*K*_d_ = 4.5 μm) and *M. smegmatis* (4 μm) (Purwantini and Daniels 1996; Bashiri et al. 2008). The *K*_m_ value for G6P is closer to that for Mtb-FGD (0.1 mm) whereas it is much lower than the equivalent value from FGD in *M. smegmatis* (1.6 mm). The observed differences in *K*_m_ values for G6P can partly be explained by different levels of G6P in these organisms; e.g., it is known that mycobacterial cells can contain high levels of G6P (Hasan et al. 2010; Purwantini and Daniels 1996).

**Table 3:** Steady-state kinetic parameters for the wild-type Rh-FGD1, K197N Rh-FGD1, K258N Rh-FGD1, and R282Q Rh-FGD1 for G6P and glucose

Rh-FGD1	Glucose-6-phosphate			Glucose		
	*K*_m_ [mm]	*k*_cat_ [s^−1^]	*k*_cat_/*K*_m_ [m ^−1^ s^−1^]	*K*_m_ [mm]	*k*_cat_ [s^−1^]	*k*_cat_/*K*_m_ [m ^−1^ s^−1^]
wild type	0.31 ± 0.016	17 ± 0.32	57,000	>300	>0.015	0.056
K197N	95 ± 12	3.8 ± 0.29	40	>300	>0.020	0.072
K258N	61 ± 5.1	0.57 ± 0.024	9.4	>300	>0.0009	0.0039
R282Q	>100	>0.047	0.67	>300	>0.0045	0.0018

**Fig. 4:**
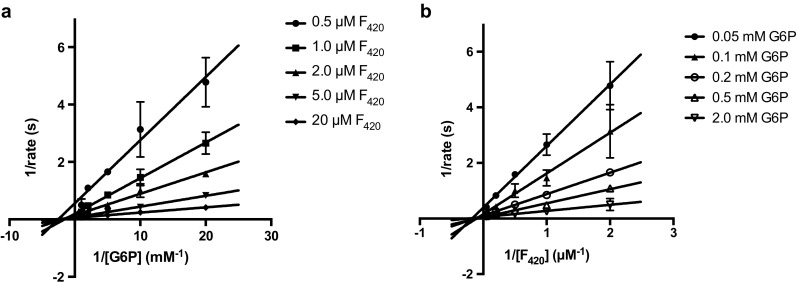
Two-substrate kinetic analysis for Rh-FGD1 via double reciprocal plots of reaction rates against **a** G6P or **b** F_420_ concentrations. These *lines* intercept at one point, corresponding to the formation of a ternary complex Rh-FGD1:G6P:F_420_ to generate 6-phosphogluconolactone and F_420_H_2_

As the FGD-catalyzed reaction involves two substrates, G6P and F_420_, we set out to decipher which mechanism is operative for Rh-FGD1, namely a ping-pong, sequential, or random mechanism. Both substrate concentrations were varied, and the F_420_ reduction rates were measured accordingly. Increasing concentrations of both substrates G6P and F_420_ resulted in an increase in reaction rates, suggesting that the reaction occurs via a ternary complex Rh-FGD1:G6P:F_420_. This is best illustrated by the observed intersection of the lines when double reciprocal values of the reaction rates and substrate concentrations are plotted (Fig. 4). Whether these two substrates bind in an ordered or a random manner, however, remains to be further investigated, e.g., by product inhibition or tracer studies with radioactive labeled substrates.

#### FGD1 overall structure

**Fig. 5:**
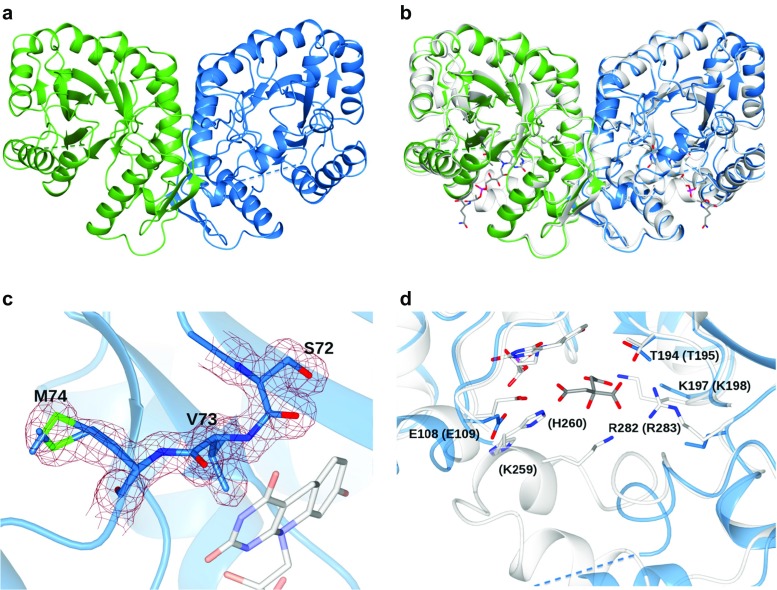
Crystal structure of Rh-FGD1 from *Rhodococcus jostii* RHA1. **a** Ribbon diagram of the Rh-FGD1 dimer showing the (α/β)_8_ TIM-barrel architecture of the two monomers colored in *light blue* (monomer A) and *green* (monomer B), respectively. The disordered region in each monomer is represented by a *dashed line* corresponding to residues 254–263 and 250–279 in monomers A and B, respectively. **b** Superposition of the Rh-FGD1 dimer (colored as in **a**) onto the homologous Mtb-FGD1 [in *white*, 84% sequence identity, PDB ID 3Y4B (Bashiri et al. 2008)] with its F_420_ cofactor bound (carbon, oxygen, nitrogen, and phosphorus atoms in *white*, *red*, *blue* and *magenta*, respectively). **c** The nonprolyl *cis* peptide bond (connecting Ser72 and Val73) and Met74 in a double conformation (sulfur atoms in *green*) are fitted to the initial 2*F*_o_ − *F*_c_ electron density map contoured at 1.2 σ (*brown chicken-wire*). As a reference, the cofactor F_420_ from the Mtb-FGD1 structure (superposed as in **b**) is drawn with *shaded colors*. **d** Close-up of the Rh-FGD1 active site superposed to Mtb-FGD1 as in **b**. The Mtb-FGD1 inhibitor citrate (carbon in *gray*) is shown bound to the active site. Putative residues involved in substrate binding are labeled with the corresponding Mtb-FGD1 residues in *parentheses*. The δ, ϵ carbon and ζ nitrogen atoms of K197, and the guanidinium group of R282 side chains were not visible in the electron density and were not included in the final model

The crystal structure of Rh-FGD1 was determined at 1.47 Å resolution by molecular replacement using Mtb-FGD1 devoid of all ligands (PDB ID code 3B4Y) (Bashiri et al. 2008) as the search model. The asymmetric unit contains two enzyme monomers forming a compact dimer (Fig. 5a), which is also observed in solution as estimated by gel permeation analysis (data not shown), similarly to the mycobacterial homolog (1.0 Å rmsd difference for 610 pairs of Cα atoms) (Fig. 5b). The very good quality of the electron density enabled us to model several residues in a double conformation and to identify a residue with a *cis* peptide bond in proximity of the active site (Fig. 5c). Only residues 254–263 in subunit A, and 250–279 in subunit B lack clear electron density and were therefore excluded from the final model. Each Rh-FGD1 monomer is comprised of residues 1–334, forming an (α/β)_8_ TIM-barrel, with the active site typically located at the C-terminus of the barrel, as observed in Mtb-FGD1 (Bashiri et al. 2008). As indicated by the Dali server (Holm and Rosenstrom 2010), this protein topology is shared also with other homologous members of the luciferase-like hydride transferase family, including a secondary alcohol dehydrogenase (Adf) and a methylene-tetrahydromethanopterin reductase (Mer) (34 and 25 sequence identity with Rh-FGD1, respectively) (Aufhammer et al. 2004; Aufhammer et al. 2005). The two Rh-FGD1 molecules present in the asymmetric unit are essentially identical, as indicated by an overall rmsd difference of 0.55 Å in Cα atomic positions of 302 residues, except for a segment comprising residues 41–49, which was excluded in the noncrystallographic symmetry restrained refinement. The dimer interface area is rather large, burying approximately 2000 Å^2^ [as analyzed by the program PISA (Krissinel and Henrick 2007)] and accounting for ~14% of the monomer’s surface. Unless explicitly stated, hereafter, we will refer to monomer A for describing the structure.

#### F_420_ binding site

**Fig. 6:**
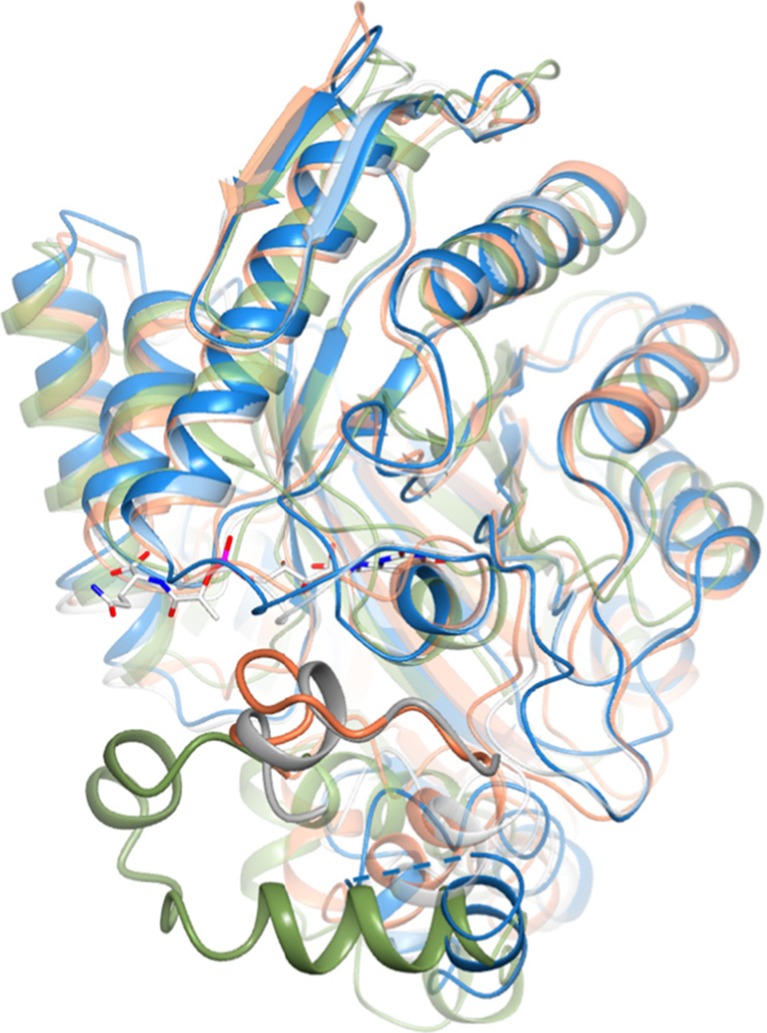
Comparison between the active site of Rh-FGD1 (*blue*) with that of Mtb-FGD1 (3B4Y, *white*), Adf (1RHC, *coral*), and Mer (1Z69, *green*). For clarity, only the F_420_ from Mtb-FGD1 is shown. The insertion regions of Mtb-FGD1, Adf, and Mer corresponding to the highly disordered segment in Rh-FGD1 (residues 254–263, represented by a *dashed line*) are highlighted in *bold style*. The orientation of the molecule is approximately 90° rotated along an axis perpendicular to the plane of the paper with respect to that in Fig. 5c. Color coding for atoms is as in Fig. 5b

All attempts to elucidate the structure of Rh-FGD1 in its holoenzyme form, i.e., with the F_420_ cofactor bound, were unsuccessful. Nonetheless, the obtained overall structure is substantially identical to that of the F_420_-bound Mtb-FGD1 and the architecture of the active site is conserved. Therefore, the F_420_ molecule was tentatively modeled in the Rh-FGD1 as a result of the superposition of the mycobacterial enzyme structure (Fig. 5b, c). In particular, the high - quality electron density clearly indicates the presence of a well-ordered nonprolyl *cis*-peptide bond between Ser72–Val73 constituent of a bulge at the end of a β strand close to the presumed binding site of the F_420_ isoalloxazine ring (Fig. 5c). This unusual *cis*-peptide is highly conserved in this enzyme family, being consistently observed in Mtb-FGD1, Adf, and Mer (joining Ser74–Val75, Cys72–Ile73 and Gly61–Val62, respectively). This bulge is essential as it serves as a backstop to hold the isoalloxazine ring from its *re*-face, bending the deazaisoalloxazine ring into a butterfly conformation (Aufhammer et al. 2005; Bashiri et al. 2008; Aufhammer et al. 2004). The F_420_ binding pocket is largely identical among FGDs, Adf, and Mer wherein the deazaisoalloxazine ring locates at the innermost part of the pocket and the hydrophilic polyglutamate tail extends into the solvent (Figs. 5d and 6). The most noticeable difference between the various structures of F_420_-binding proteins is a helical coil region located at the C-terminus of the TIM-barrel, creating a sort of lid element that stabilizes cofactor binding (Fig. 6). In Rh-FGD1, the sequence for this structural element is shorter than that of the homologous enzymes and corresponds to the disordered part (residues 254–263 in monomer A). The high flexibility of this region may correlate with a more dynamic interaction with the cofactor and may well explain the difficulty in obtaining the Rh-FGD1 structure in its holoenzyme form.

#### Glucose-6-phosphate binding site

In Mtb-FGD1, a citrate molecule, most likely derived from the crystallization solution, was found to bind adjacent to the F_420_ molecule and later proved to be a competitive inhibitor for Mtb-FGD1. Citrate occupies a cavity with a size that can fit G6P in an orientation that is suitable for catalysis. This allowed the modeling of G6P into the active site of Mtb-FGD1, revealing highly conserved residues involved in substrate binding and catalysis (Bashiri et al. 2008) (Fig. 5d). It has been postulated that in Mtb-FGD1, the phosphate moiety of G6P occupies a positively charged pocket constituted by side chains of Lys198, Lys259, and Arg283 (corresponding to Lys197, Lys258, and Arg282 in Rh-FGD1, respectively) (Bashiri et al. 2008) (Fig. 5b). In Rh-FGD1, out of the three residues, the position of Lys258 is unknown as it is part of the disordered region. Instead, Lys197 and Arg282 are visible and adopt a similar conformation with respect to the corresponding residue in Mtb-FGD1. Nevertheless, a part of their side chains lacks clear electron density (Cδ, Cϵ, and Nζ of the former and the guanidinium group of the latter), which indicates a much higher flexibility. Sequence alignment indicated that the three residues are strictly conserved in proteins exhibiting FGD activity (Bashiri et al. 2008). Rv0132c—sharing 36% sequence identity with Mtb-FGD1 and previously annotated as Mtb-FGD2—does not contain these phosphate group binding residues and consistently showed no such assigned activity (Bashiri et al. 2012). In fact, Rv0132c was later proven to be an F_420_-dependent hydroxymycolic acid dehydrogenase and was proposed to be an unprecedented antitubercular target that may also be inhibited by the novel drug pretomanid (PA-824) (Purwantini and Mukhopadhyay 2013).

To further probe the roles of these residues, we generated single mutations, namely K197N, K258N, and R282Q Rh-FGD1. In comparison to the wild-type Rh-FGD1, the mutants showed a drastic decrease in catalytic efficiency for G6P, as indicated by *K*_m_ values of two orders of magnitude higher than that of the wild-type enzyme (Table 3). The rate of catalysis was also considerably affected: The R282Q mutant virtually lost activity whereas the K197N and K258N mutants had a 4.5- and 30-fold lower *k*_cat_ value, respectively, compared with the wild type. Disruption of the phosphate binding pocket may, to a certain extent, also affect the substrate specificity. In fact, when glucose was used as a substrate instead of G6P, the mutant K197N showed a slightly improved *k*_cat_/*K*_m_ when compared with the wild-type enzyme (Table 3). These data unequivocally verified that the three targeted residues are crucial for the binding of the phosphate moiety of the G6P. Moreover, it might become possible to improve FGD activity towards glucose, e.g., by random mutagenesis of residues forming the G6P binding pocket.

## Discussion

### Physiological role of Rh-FGDs

F_420_ is an unusual redox cofactor originally found exclusively in a restricted number of microbes, such as archaea and actinomycetes. Astonishingly, a bioinformatics study in 2010 indicated that F_420_ can be much more widespread than previously thought and present in 11% of all sequenced bacteria and archaea (Selengut and Haft 2010). In particular, *R. jostii* RHA1 was predicted to contain at least 104 deazaflavoenzymes, an impressively large number. In line with this prediction, we present here the first experimental evidence for the presence of F_420_-dependent enzymes in *R. jostii* RHA1. More specifically, the *R. jostii* RHA1 genome encodes at least three FGDs: RHA1_RS43115 (WP_011600337.1), RHA1_RS10755 (WP_011595003.1), and RHA1_RS43570 (WP_011600440.1) (referred to as Rh-FGD1, Rh-FGD2, and Rh-FGD3, respectively). We have focused our exploration on Rh-FGD1, the best expressed one, characterized the kinetic properties and elucidated the structure of the apo protein at high resolution. On a cautionary note, it should be noticed that the Rh-FGD1 and Rh-FGD3 are plasmid encoded whereas Rh-FGD2 is instead encoded by a chromosomal gene. Preliminary tests (data not shown), however, indicated that Rh-FGD1 and Rh-FGD2 have comparable specific activity. This gene redundancy is generally believed to facilitate the high catabolic versatility in rhodococci (van der Geize and Dijkhuizen 2004; McLeod et al. 2006).

F_420_-dependent glucose-6-phosphate dehydrogenase has been suggested to be the primary enzyme responsible for the F_420_ reduction in several actinomycetal genera, including mycobacteria, thereby linking their central metabolism to the F_420_ reduction reaction (Purwantini and Daniels 1996). The main role of mycobacterial FGDs appears to be the generation of F_420_H_2_ as these bacilli also encode the conventional NADP^+^-dependent FGDs (Purwantini et al. 1997), which interestingly showed no significant phylogenetical relation to FGDs (Purwantini and Daniels 1998). A deletion of either *fgd* or *fbiC*—a gene involved in the biosynthesis of F_420_—renders these mycobacterial strains incapable of reducing xenobiotics via F_420_H_2_-dependent reductases (Taylor et al. 2010; Hasan et al. 2010; Stover et al. 2000; Manjunatha et al. 2006). Nevertheless, the physiological role of FGDs in *Rhodococcus* spp. remains largely unclear. It is well known that in *Rhodococcus opacus* and *Nocardioides simplex*, the reduced F_420_ is supplied mainly by F_420_:NAPDH oxidoreductases (FNOs) rather than FGDs (Ebert et al. 1999; Heiss et al. 2002; Ebert et al. 2001; Heiss et al. 2003). FNOs were found to be expressed from the same operon as the F_420_H_2_-dependent hydride transferases, which are responsible for the degradation of environmental nitroaromatic compounds such as picrate and 2,4-dinitrophenols (Ebert et al. 1999; Heiss et al. 2002; Ebert et al. 2001). However, it cannot be excluded that FGDs also play a (crucial) role in generating F_420_H_2_. As very little is known about the natural substrates of the F_420_H_2_-dependent enzymes in *Rhodococcus* spp., it can be speculated that FGDs are primarily responsible for providing the reductant for the endogenous metabolism, maintaining the redox homeostasis during normal growth or in response to oxidative stress as observed in mycobacteria. Several lines of evidence have revealed the pivotal role of G6P as an electron reservoir mobilized via FGDs in protecting mycobacteria against oxidative and nitrosative stress (Hasan et al. 2010; Gurumurthy et al. 2013). In fact, the presence of a NADP^+^-dependent FGD alone failed to render a *M. smegmatis* mutant deficient in FGD capable of surviving oxidative stress. Further investigations, e.g., gene deletion studies in combination with isotopic labeling metabolomics, are therefore necessary to decipher the precise role of FGDs in rhodococci (van der Geize et al. 2008).

### FGDs as biocatalyst for cofactor regeneration

The biocatalytic reduction of F_420_ has been carried out so far with the use of Mtb-FGD1 (Manjunatha et al. 2006). Such reduced F_420_ is essential in studying deazaflavin-dependent reductases. However, mycobacterial FGDs are poorly to moderately expressed as soluble protein when *E. coli* is used as a heterologous expression host (Purwantini and Daniels 1998; Bashiri et al. 2007; Manjunatha et al. 2006). To overcome this limitation, a dedicated *M. smegmatis* expression strategy was developed to enhance the solubility of mycobacterial proteins. The typical yield obtained was 7 mg of pure recombinant Mtb-FGD1 from 1 L of *M. smegmatis* culture (Bashiri et al. 2007). In contrast, we produced soluble Rh-FGD1 in rather high yield: 80 mg of pure protein L^−1^ of culture. The developed *E. coli*-based expression system facilitates the routine production of soluble FGD which can be used for the synthesis of reduced F_420_. Rh-FGD1 is a relatively fast enzyme, with a *k*_cat_ of 17 s^−1^ for G6P (Table 3). In addition, Rh-FGD1 appears to be thermostable in most common buffers and additives (Fig. 3); upon storage at −80 °C, Rh-FGD1 retained >90% activity after 1 year. The observation that Rh-FGD1 displayed some activity, yet very low (Table 3), with glucose, a much cheaper substrate instead of G6P, hints to the possibility to engineer Rh-FGD1 into a more efficient F_420_-dependent glucose dehydrogenase. The first logical target for such tailoring efforts would be the phosphate binding pocket. Interestingly, when glucose was used as substrate, the mutant K197 N showed an improved *k*_cat_/*K*_m_ value of 30% higher than that of the wild type. Therefore, by fine-tuning these residues by site-directed mutagenesis, one could obtain mutants with improved activity with the cheap cosubstrate glucose. Given its robustness and accessibility, Rh-FGD1 represents a potential candidate for the biocatalytic reduction of F_420_ in larger scale or in fusion with other valuable F_420_H_2_-dependent reductases in a redox self-sufficient whole-cell biotransformation.
